# Pioneer Legislation on Second Order of Sexual Harassment: Sociolegal Innovation in Addressing Sexual Harassment

**DOI:** 10.1007/s13178-021-00571-0

**Published:** 2021-03-26

**Authors:** Ana Vidu, Gema Tomás, Ramon Flecha

**Affiliations:** 1 Av de las Universidades, 24, 48007 Bilbao, Spain; 2 Diagonal Avenue, 690, Office 4100, 08034 Barcelona, Spain

**Keywords:** Sexual harassment, Second order of sexual harassment, Bystander intervention, Legal innovation, SOSH legislation, Second order violence

## Abstract

**Backgroud:**

Countless efforts to combat sexual harassment have been proposed, and for the first time in history, the second order of sexual harassment (SOSH) has been legislated under the term second-order violence (SOV) by a unanimous vote of the Catalan Parliament. Advances in preventing and responding to sexual harassment contribute to highlighting the intervention as being crucial to supporting survivors against retaliation. A lack of support provides a general explanation on why bystanders tend not to intervene and highlights the reality that reprisals are suffered by those who support victims.

**Methods:**

From the existing knowledge about sexual harassment prevention and response mechanisms, this paper analyzes scientific evidence through a review of the literature published in databases, as well as legislation, reports, and other materials.

**Results:**

The context that enables SOV legislation is grounded in three realms: (1) bystander intervention and protection, (2) the role of support networks in protecting survivors, and (3) awareness and legislation of SOSH. An active bystander refers to the involvement of someone who is aware of potential sexual harassment situations.

**Conclusions:**

The lack of legislation against SOSH limits bystander intervention and support; therefore, legislating protection for supporters has become urgent and necessary. Legislating SOSH has great social implications because gender equality cannot be fully achieved if bystander protection is not legally considered. Policy Implications: As no legal system has previously contemplated SOSH, its pioneering parliamentarian approval and establishment by Catalan law constitute a legal key innovation for the field of gender and women’s studies. In fact, evidence reported here are important in developing further regulations and policy.

**Policy Implications:**

As no legal system has previously contemplated SOSH, its pioneering parliamentarian approval and establishment by Catalan law constitute a legal key innovation for the field of gender and women’s studies. In fact, evidence reported here are important in developing further regulations and policy.

## Introduction

Gender-based violence (GBV) still constitutes a challenging reality in our society, and efforts to overcome it are continuously increasing (UN Women, 2018). In its last report, the World Health Organization (WHO, 2017) estimated that the number of women around the world who have suffered some type of physical or sexual violence throughout their lives is 35% (1 in 3). Indeed, the victims’ age is constantly decreasing, with 30% of women between 15 and 19 suffering or having suffered gender-based violence in their sexual affective relationships (WHO, 2017). The organization considers violence against women in its broadest aspects, particularly sexual violence, as a major violation of women’s human rights. The existence of GBV also means a violation of the right to science, which was established in Article 27 of the Declaration of Human Rights of the United Nations (UN, 1948), which states the right not only to participate in scientific progress but also in the benefits derived from it.

Violence against women is also a great challenge in different spheres, such as universities, companies, educational and political environments, and during leisure time or nightlife activities. Research has shown that sexual violence should be addressed from different aspects since it also occurs in several spaces and at the workplace (Richman et al., 1999). Undoubtedly, we are in a historical social moment considering not only the recognition of GBV across social spheres but also the intervention approaches and improvements (National Academies of Sciences, Engineering, and Medicine, 2018). Society is asking for solutions that can improve the lives of many members (Torras-Gómez et al., 2019). Drawing on this, efforts have been made to achieve efficient mechanisms to prevent and respond to this problem.

Evidence has shown that bystander intervention (Banyard et al., 2005), the active support provided by those who are aware of a potential harassment case, even if not of the direct victims, constitutes one of the most efficient measures to overcoming sexual harassment (Coker et al., 2016). However, bystanders may also suffer from injury and retaliation by taking the side of the survivors, which is defined as bystander harassment (O’Connor, 1999) or second-order sexual harassment (Dziech & Weiner, 1990). Thus, support for bystanders is essential for overcoming violence against women.

Christopher O’Connor (1999) emphasized the existing legal support in the USA for *bystander injury* under Title VII of the Civil Rights Act of 1964 (employment anti-discrimination law), in which the Supreme Court also recognizes hostile work environment claims. Arguably, this perspective on potential injuries to a bystander could be considered based on the need to offer protection against harassment that does not occur directly. The Obama Administration reinforced Title IX Legislation^1^ through strong policies specifically for approaching sexual violence on college campuses (Cox, 2018; Rosenfeld, 2008). Considering the evidence obtained thus far, networks and student movements have achieved the greatest advances in tackling this problem on institutions and college campuses (Clark & Pino, 2016). Thus, student struggles have shed light on the problem and pushed for the creation of protocols and legislation to address and prevent gender-based violence. Such work has had an enormous effect on increasing the number of cases reported but has identified other areas where GBV must be considered and addressed. Thus, the second order of sexual harassment (hereafter SOSH) is particularly germane to the college context in which it first emerged and developed prior to application in other contexts.

Although actions to prevent and respond to sexual violence have been widely addressed over recent decades, the role of SOSH in overcoming gender violence has not yet been sufficiently explored or legislated. The driver of this research was to provide a sociolegal response to the need to overcome GBV via SOSH legislation in different spheres, and our aim was to analyze the extent to which the three realms (bystander intervention and protection; networks of support; awareness and legislation of SOSH) have an effect in the different contexts studied.

While reasons in favor of legislation are presented to empower survivors, one of the main objectives of this article is that to support direct victims and victims of SOSH, not only morally but also legally, the first legislation for SOV and the importance of properly approaching GBV must be promulgated. The secondary goals for supporting this main objective are to provide arguments on why SOSH legislation will contribute to building institutions and organizations free of sexual violence to increase social equality and overcome GBV, which are the strongest reasons supporting this legislation. These aims consist of (1) raising greater awareness about bystander intervention and protection, (2) analyzing the role of support networks for protecting survivors, and (3) discussing SOSH legislation’s impact on the defense and empowerment of survivors. At the same time, this pioneering norm and the goals of the analysis are intended to introduce SOV into the scientific agenda.

## Mapping the Field

One in 5 women are sexually assaulted in US colleges every year (Krebs & Lindquist, 2014). One in 3 female undergraduates and 1 in 5 male students have reported that they told by a friend that they were the victim of an unwanted sexual experience (Banyard et al., 2010). This reality is rarely reported (García-Hernández et al., 2020); indeed, 88% of women sexually assaulted on university campuses do not report their assault (KFF, 2015). According to the Association of American Universities, 27.2% of university women have experienced some type of undesirable sexual contact, from touching to rape, since their enrollment in college (AAU, 2015). This alarming scenario causes serious consequences for victims (Katz et al., 2011). The World Health Organization considers GBV a “global health problem of epidemic proportions” (WHO, 2013) that affects the health of physical, mental, sexual, and behavioral women (Preiser & Assari, 2008). Studies and institutional mechanisms tend to increasingly encourage victims to report (Grauerholz et al., 1999), while protection for those who support victims is still an underexplored matter. At this point, the lack of support and the lack of networks of support tend to be common experiences for survivors (Sable et al., 2006).

Understanding sexual harassment in its broadest sense has involved analyzing SOSH and its implications for victims and their communities (Flecha, 2021). Coker et al. (2016) evaluated the impact of the results of several university measures and mechanisms that have been implemented to address sexual harassment. Through the first multiyear empirical evaluation of the 2013 Campus Sexual Violence Elimination Act (which requires US colleges to provide bystander-based training to reduce sexual violence), they demonstrated that programs based on *bystander intervention* are some of the most efficient programs for addressing sexual harassment and reducing GBV. To prioritize direct victims, the reality of SOSH must be approached. Although bystander training is effective (Coker et al., 2016) and individuals can learn to respond to a potentially damaging situation or interaction to positively influence the result, if there is no protection for them, then people will tend to not intervene, even if they know how to do so and are trained for it. To convert a passive bystander into an active bystander, protection and legislation become crucial. The current legislation on second-order violence contributes to creating greater awareness about the protection for those who dare to support survivors as well as on their essential role in the entire process of protecting direct victims. There is an agreement on the need to take action against sexual harassment, and the pioneering legislation presented here embodies one of the most important steps in the advancement of survivor protection and GBV prevention.

The first known definition of SOSH, which was applied to college campuses, was published by Dziech and Weiner (1990) as historical evidence of the need to implement mechanisms to overcome GBV and the serious effects it entails for victims’ supporters. According to the authors, the second order of sexual harassment is defined as follows: “Sexism on campus creates a second order of sexual harassment victims, those who advise, support, and rule in favor of the primary victims. These are the affirmative action officers, ombudspersons, counsellors, assistant deans – the people assigned, and usually committed, to helping sexual harassment victims” (p. XXIX). Usually, they are not direct victims of GBV but *bystander victims* because of their solidarity with other victims, so they were called second-order victims. Dziech and Weiner shed light on a crucial issue at that moment by broadly discussing the consequences of harassing victims and their environments. However, subsequent evidence showed that individuals in positions where they can protect victims (such as officials) are not necessarily on the victims’ side. Therefore, being a bystander does not imply providing support. Sometimes, informal mechanisms are more effective for providing support than formal ones (Reilly et al., 1992). However, both spheres are connected. Because informal mechanisms work, they need to be formalized so that they have the legitimacy to act. Thus, support needs to be formalized to ensure the safety of victims.

In 2017, the concept of SOSH was published for the first time in Spain and defined as follows: *physical and/or psychological violence against persons who support victims of sexual harassment. Some people, groups and institutions that support survivors become subject to violence when they accompany victims in the process of reporting or when they defend victims from revictimization as a form of coercion against such support* (Vidu et al., 2017). This definition of SOSH pretends to recreate its meaning in the current reality while filling the gap produced during these decades in the scientific community, where the SOSH concept has been poorly considered and the “survivors first” concept (Choate, 2003) was primarily examined. Because survivors are the primary victims, their support needs to be protected, which is why we embrace the discover of new insights on SOSH, such as by its legislation as SOV.

## Second-Order Harassment as a Legislative Innovation

The SOV regulation is configured as the new Catalan law^2^ and addressed here as a new category of harassment that legally arises when the person suffers some kind of damage because of assisting and protecting those who suffer sexual harassment. Neither the European Directives that incorporated the definitions of harassment^3^ nor the state regulation of transposition conducted through *Organic Law 3/2007 of March 22 for the effective equality of women and men*^4^ explicitly incorporated this legal category of harassment, nor did they mention the legal attention warranted for those who consciously and voluntarily decide to assist, defend and protect those who may go through situations of this nature. Therefore, after carefully searching among regulations and legal documents, the Catalan law constitutes the first time ever that the second order of sexual harassment has been legislated.

The second order of sexual harassment legally recognizes whoever becomes a victim of harassment by carrying out active behavior aimed at defending and intervening in favor of the direct victim of sexual harassment. This Catalan norm takes this legislative step to make visible the frequent situations in which those who act on behalf of the victim of harassment also suffer harassment (defined as “second order”), either from the same harasser or from a different person. This visibility will undoubtedly contribute to the eradication of harassment, which is supported by scientific evidence provided in this article (Madrid et al., 2020; Pinchevsky et al., 2020).

European Directives of the European Parliament and The Council (Official Journal of the European Union, 2006) have broadly defined sexual harassment. Article 2.1 of the 2006/54/EC Directive defines a “victim of harassment” as someone who directly suffers as well as any person who is “considered injured.” As sexual harassment is the typified concept, the condition of who is considered “victim” is not yet configured under any legal framework currently existing. Therefore, victims could be direct or second-order victims, thus involving the consideration of second-order harassment. Legally, the causal link between direct sexual harassment and second-order sexual harassment implies the need to prove the former so that the latter occurs. If such a causal link is legally considered, then the proof of the damage and the right to be repaired will be more achievable.

For the legal construction of this category, first, it is necessary to start from the definition of harassment in the European Directives mentioned above. Both accept the European meaning of “harassment” as “the situation in which unwanted behavior related to the sex of a person occurs with the purpose or effect of undermining the dignity of the person and creating an intimidating environment, hostile, degrading, humiliating or offensive.” Similarly, the specific definition of “sexual harassment” is defined as “the situation in which any unwanted verbal, nonverbal or physical behavior of a sexual nature occurs with the purpose or effect of undermining the dignity of a person, in particular when it is created an intimidating, hostile, degrading, humiliating or offensive environment.” In this vein, the Spanish Law on effective equality in Article 7 establishes the following in its first section: “Without prejudice to the provisions of the Penal Code, for the purposes of this Law, any behavior, verbal or physical, of sexual nature that has the purpose or produces the effect of undermining the dignity of a person, in particular when an intimidating, degrading or offensive environment is created.” It includes the following in its second section: “Any behavior carried out based on a person’s sex, with the purpose or effect of undermining the dignity of that person and creating an intimidating, degrading or offensive environment constitutes harassment on grounds of sex.”

Based on these legal meanings of harassing behavior, the classification of “second order of sexual harassment” constitutes a legal innovation, both in the objective scope of harassment situations, which are formally expanded through this conduct classified as “second order,” as well as in the subjective determination of a “victim” of harassment. In this sense, broadening the scope of whoever is considered a “victim” by incorporating someone who is a “second-order” victim deserves attention because it goes beyond what the Spanish legal system considers as a victim of a criminal offense. According to the statute of the victim regulated in Law 4/2015 of April 27 and the statute of the crime victim^5^ in Article 2, a direct victim is considered: “any human being who has suffered damage or harm to his or her own person or property, especially physical or mental injuries, emotional damage or economic damage directly caused by the commission of a crime” and an “indirect” victim is considered the spouse, partner and children, parents and other relatives in the established terms^6^.

This concept of “victim” is a transposition of Directive 2012/29/EU of the European Parliament and of the Council of October 25 (EU, 2012), which establishes minimum standards on the rights, support, and protection of crime victims^7^. This European norm considers “victims” in Article 2.1, both as (1) the human being who has suffered damage or harm, especially physical or mental injuries, emotional damage or economic damage, directly caused by a criminal offense and (2) the relatives of a person whose death has been directly caused by a crime and has suffered damage or harm as a result of the death of that mentioned person.

However, as authors such as García Rodríguez (2016) mentioned, this concept of “victim” in Spanish legislation and that of *victim of* violent crimes and crimes against sexual freedom as well as the concept contemplated in the European Directive should be broader and follow the criteria of the United Nations, which includes the family members or dependents of the direct victim as well as those who may have suffered any type of damage when intervening to help or try to prevent the crime, e.g., “good Samaritans”^8^. All measures focus on the victim, recovery, care, prevention but do not extend protection to potential defenders. All this evidence is pioneering, although the concepts currently recognized under SOV legislation have not been thoroughly considered.

## Pioneering Spanish Case to Better Present SOSH

After decades of institutional silence, in 2011, approximately fourteen victims went through the long and difficult university administration process for complaining against the most well-known and recidivist professor in the country. The process lasted approximately 2 years until it achieved the prosecutor stage (Puigvert et al., 2017). The survivors of this case had received essential support from few but crucial professors. Actually, it was one full professor who filed this pioneering complaint in the name of the first survivor complainant who did not dare to do it by herself. All the supporters of this case received some kind of defamatory attack and criticism (Valls et al., 2016). The Equality Commission of the university at that time was not only in charge of “protecting” the institution, thereby placing itself on the side of the harasser, but they exercised different types of reprisals to silence the victims, including victimization towards them and their supporters. Because second-order protection was not legalized at that time, they had no protection.

The supporters’ support was crucial during the process, especially for legal service interrogations led by legal instructors who are used to blaming survivors. Once the internal process was exhausted, the complaints reached the Public Prosecutor’s Office once the facts had already been prescribed, precisely because of the internal “process.” Actually, the fact that the internal process was so long, hunted the University intention for the case to be filed by the Office of the Prosecutor because of the statute of limitation. Finally, it happened and the case was filed. The prosecutor’s office report was in favor of the survivors since it clearly stated that sexual harassment had occurred, including a quote from the dean admitting that she was aware of the acts committed by the accused professor since she was a student herself. The press reported the case, and several media outlets highlighted the voices of the victims (RTVE, 2017), who become survivors thanks to the support they had from different spheres.

A previous and pioneering event in Spain to publicly discuss SOSH was held in Barcelona in December 2016 under the framework of the first workshop on the second order of sexual harassment”^9^. Five roundtables were conducted during the workshop, and they focused on different areas where SOSH may occur. There were people representing different political parties and members of social organizations, the media, the educational field, and the legal field. During the workshop, the need to address this problem was openly recognized, which only hours earlier represented a reality that had never been named. From different areas, participants identified environment situations as second-order harassment, and they considered it crucial to tackle such harassment to end GBV. Therefore, debates conducted in that pioneering workshop emphasized the link between overcoming SOSH and erasing direct violence, which implies that the second cannot be overcome if the first is not properly addressed.^10^

## Methods

Many measures from different perspectives have been dedicated to the prevention and overcoming of one of the main types of social discrimination: gender-based violence. To properly address the concept of SOSH, which is currently included in the legal spectrum, we implemented an exploratory interpretative approach focused on a systematic, theoretical, normative, and conceptual literature review.

### Literature and Analytic Review

We performed an analysis of the existing scientific literature on the subject using scientific databases, such as *Web of Sciences*, *Sociological Abstracts*, and *Journal Citation Reports*. From the extensive information on GBV, we selected evidence either that made reference directly to SOSH as a concept or that exposed reprisals suffered by people who acted as defenders of primary victims. For this research, the authors also performed a study of the current discourse via webpages, media, and newspapers. On the basis of the analysis, discourses and demands on university students’ mobilization in relation to sexual violence on college campuses were considered. Movements that occurred in the USA that emphasized the historical struggle also inspired similar student movements in other places a few decades later (Puigvert et al., 2017). In the same vein, social mobilization leads to legal avenues in reality and creates new social policy.

### Data Collection

Conducting desk research, the authors reviewed the following data: (a) prevailing regulations and legislation on GBV to identify their content regarding this reality within the academic context. The authors also analyzed (b) existing law by including the study of judicial sentences that especially addressed the categories of the second order of sexual harassment or second-order violence, among others, which includes national and international databases of jurisprudence; and (c) constitutional courts at the European level, such as the *European Court of Human Rights* and the *Court of Justice of the European Union *(*CJEU*), as well as *United Nations documents* and *directives*. While it is still an open debate among the scientific community on how sexual offence trials should contemplate and protect complainants’ and defendants’ rights (Leahy, 2014), what is still fully underexplored is the protection for those who support survivors and may suffer reprisals because of it.

### Data Analysis

A snowballing strategy was employed to identify other sources in addition to those already presented. For example, in selected sources referring to specific legislation, the authors searched for data extended to other reports and online available information on sexual harassment and victim protection. Secondary data already presented by previous research and legal databases were collected for this particular research. The review focused on some disciplinary fields, including sexuality research, education and humanities, sociolegal and socioeconomic studies, law, political science, sociology, psychology, media, and feminist studies.

For the data analysis, some classification divisions have been established according to the categories from the literature review. These categories embrace the following: (1) current literature on SOSH and its consequences for victims and their supporters, (2) documentation on support networks protecting survivors, and (3) pioneering legislation that leads to overcoming violence by protecting second-order victims. Finally, the social policy implications of SOV legal regulations will be discussed. In the elaboration of the mechanisms through which social struggles come to influence legislation, a comparative law analysis was performed by observing the normative framework and current laws within different countries and comparing among them the measures applied for addressing SOSH.

## Scenarios in Which SOSH May Occur

Universities constitute the space in which the concept of second-order sexual harassment emerged, and such phenomena have also occurred beyond universities in several contexts and situations. As reported, GBV occurs at early ages, and children may be highly exposed to violence in cultures where silence and disbelief predominates (Jeremiah et al., 2017). Some children who dare to complain about violence or abuse experience a lack of trust, which discourages them from coming forward. The lack of support among the social network of the victim perpetuates situations of violence (Reilly et al., 1992). The literature has already shown that silence is maintained because the number of people who dare to provide help to stop the situation is rather low; in fact, the culture of silence inhibits prevention (Jeremiah et al., 2017). Indeed, children’s exposure to violence also occurs in the educational environment (UN, 2016). For instance, when two children fight, the intervention of a third person is key to ending that argument. However, this intervention is subject to protection. Therefore, schools have the potential to break that silence and implement mechanisms of child abuse prevention and intervention. Child violence during the severe confinement implemented due to the COVID-19 outbreak has also been acknowledged. The elements required to overcome such vulnerability are driven by promoting alternatives that protect children from these realities and make them feel as if society is on their side and that they will never run out of support (Roca et al., 2020). Proper measures to protect all victims are urgent and necessary.

SOSH also occurs in other areas worldwide, such as the press (Madrid et al., 2020), social organizations (Smith et al., 2020), or at the workplace (Prado et al., 2020; Chen & Chu, 2020), and such actions include the revictimization that happens for survivors (Stephens & Eaton, 2020) and second-order victims. In a newspaper article^11^, Melgar reports on three cases of people who quit their jobs because of harassment, with one at a university, one at a company, and the third at a nightlife establishment. All of them are relevant to the issue we are addressing here.

There is also increasing literature on sexual abuse during leisure activity (Hartill & Lang, 2018) and violence in leisure spaces, such as boy scouts or in discos (Duque et al., 2020). All these spaces share the potential social awareness that inappropriate actions are happening but the associated members choose to not intervene. As described, harassment can happen in different spaces, but what all harassing situations do have in common is that active intervention is need, which will increase due to the protection of bystanders. A regulation of SOSH will mean progress in all these areas and therefore in society as a whole. It would mean protection for all those who felt abandoned after daring to support someone against harassment, coercion, or complaints.

## Awareness of SOSH Reprisals for Victims and Their Supporters

Many students do not report sexual harassment because of possible backlash, negative comments, and fear of not being believed. Being aware of how difficult it is to report, victims do not dare to take this path if they are not strong enough to endure potential victim blaming, which has a silencing effect on survivors (Fisher et al., 2000). Revictimization (or double victimization) is understood as a situation in which the consequences already suffered by the victims are being multiplied, causing her/him to feel fear and even guilty about the situation. In this sense, the struggle is to persuade victims to report and break the silence as well as to have students willing to talk about this issue. Therefore, most university mechanisms are directly focused on students telling their stories (Bird, 2002; Racionero et al., 2018). In the same way, secondary victimization, victim blaming, and discrimination (Jackson et al., 2017) create a message for other victims to prevent them from denouncing harassing behavior because it will make the situation worse. One of the challenges of our century consists of providing evidence-based solutions to overcoming this victimization, especially for the most vulnerable communities (Serrano & Rios, 2019). To overcome this effect and generate supportive responses, two aspects need to be addressed: (1) success for first- and second-order victims and (2) laws that defend those who support survivors. In this vein, this article is making progress. The role of laws and policies on specific topics related to gender and equality has contributed to advances in mechanisms for overcoming sexual harassment (Pineiro & Kitada, 2020).

## Role of Support Networks in Protecting Survivors

Extensive literature has been published on social movements and their power of transformation as well as student movements and their capacity to generate university improvements at different levels (Steinberg, 1991; Reid & Dundes, 2017). Current law during the late 1980s and early 1990s, mechanisms, orientation guides, programs, and specific offices were developed at North American universities as a result of student campaigns against harassment, and the role of student support groups would later be recognized by scholars (Gold & Villari, 2000; Freedman, 2013).

The driving force behind each action, complaint, or mechanism against sexual violence is solidarity, which also extends beyond universities (Madrid et al., 2020). Solidarity and support frame every story told by victims, activists, faculty, or administrators (Valls et al., 2016). Because sexual violence in academia is a community problem, it involves everyone, thus making networks of solidarity necessary. The study of Banyard et al. (2010) demonstrated that most women who are assaulted, 66% to 87% according to Ullman et al. (2008), disclose their situations to a friend or to someone to whom they feel close or trust. Therefore, many campaigns aim to address this type of situation: how to treat a friend and how to respond to someone describing abuse. Relying on peer support prevents or helps survivors face victimization (Reilly et al., 1992).

University programs that combat harassment are designed to prepare organizations and communities to implement strategies of prevention. They achieve this goal both by developing information on the subject (such as research) and by providing training to professionals who implement appropriate measures for mobilizing witnesses of harassment (bystander intervention). Student and faculty movements across some US college campuses, for example, have already had an impact on the political agenda and the reformulation of laws. The mobilizations in which the creators of the *End Rape on Campus Association* were involved were considered by the US president and his government, thus leading to the establishment of a specific Task Force to address this problem as well as specific mechanisms, such as the “It’s on US” and “Not Alone” campaigns (US Government, 2017).

## Concept of Second-Order Harassment Victims from an International Legal View

As mentioned, previous laws did not recognize someone who provides assistance, defense, or protection to the victim as a potential victim. The recently approved Catalan law echoes the Basic Principles and Guidelines on the Right to a Remedy and Reparation for Victims of Gross Violations of International Human Rights Law and Serious Violations of International Humanitarian Law, which are included in Resolution 60/147 approved by the General Assembly on December 16, 2005 (United Nations Human Rights, 2005)^12^. These 27 principles apply to gross violations of international human rights norms and serious violations of international humanitarian law, which due to their very serious nature constitute an affront to human dignity. These principles adopt a victim-oriented approach, and the international community affirms its human solidarity with victims of violations of international law.

Article V (*Victims of gross violations of international human rights law and serious violations of international humanitarian law*) of this UN Resolution includes two topics that become the crucial basis for the issue addressed in this article. Thus, Principle 8 refers to the “Victims of manifest violations of international human rights norms and serious violations of international humanitarian law” in the following terms: *For purposes of the present document, victims are persons who individually or collectively suffered harm, including physical or mental injury, emotional suffering, economic loss or substantial impairment of their fundamental rights, through acts or omissions that constitute gross violations of international human rights law, or serious violations of international humanitarian law*. Where appropriate, and in accordance with domestic law, the term “victim” also includes the immediate family or dependents of the direct victim and persons who have suffered harm in intervening to assist victims in distress or to prevent victimization 13. In view of the final statement of this principle, the term “victim” is broad because direct victims and indirect victims (family members and dependents) include those people who have suffered damage by “intervening” to provide “assistance” to victims in danger or to prevent victimization. Accordingly, Principle 9 of this resolution considers an extension of the concept of the direct victim to also include those people who have suffered damage when intervening to assist victims in danger or to prevent victimization: *A person shall be considered a victim regardless of whether the perpetrator of the violation is identified, apprehended, prosecuted, or convicted and regardless of the familial relationship between the perpetrator and the victim).*

These principles were drawn up by the Dutch jurist Theo van Boven^14^, special rapporteur for the United Nations on the right to reparation for victims of serious human rights violations from 1986 to 1991, and after more than 20 years of research, they were finally adopted in the General Assembly. They are not binding on the Member States, but they are a guide that helps the states in the implementation of public policies. These principles are considered the first codification of the rights of victims of human rights violations to reparation for the damages suffered and access to justice in accordance with the respective national legal systems.

Those who help victims, e.g., lawyers and doctors, are frequently the target of abuse. This international legal declaration takes into account the context in which these types of serious human rights violations take place: lawyers who suffer harassment by telephone, apparently “wrong” kidnappings, physical and mental abuse, actions to discredit their professional work, etc.^15^ In the international arena, a process of broadening the concept of a “victim” has been observed in recent decades to include family members. An expansion in cases of serious human rights violations should be considered a step forward in the recognition of the effects that these violations have on people other than direct victims^16^.

The system of the Inter-American Court of Human Rights support the idea that highlights the legal importance of taking into account those who protect the victim and prevent revictimization. This focus on the victim confirms the importance of considering second-order harassment, which is precisely based on this perspective both to protect the victim and to contribute to the eradication of the unlawful act of harassment itself. The Council of Europe Convention on preventing and combating women and domestic violence approved in Istanbul on May 11, 2011 (BOE number 137, June 6, 2014) does not include the need to legally consider who helps to women victims of gender violence who are threatened or otherwise harassed.

## SOSH Novel Contribution

On December 18, 2020, different political positions of the Parliament of Catalonia agreed to introduce the concept of second-order violence in the amendment of *Law 5/2008, of April 24, on the right of women to eradicate sexist violence*. In this way, Law 5/2008 incorporates a significant novelty by including violence in the social or community sphere (Art. 5.4), the so-called second-order violence^17^. This harassment has been legally defined as physical or psychological violence, retaliation, humiliation, and persecution exercised against people who provide support to victims of sexist violence. Acts that deter the prevention, detection, care, and recovery of women in situations of sexist violence are included.

The visibility provided for this type of harassment is a legal advancement worth mentioning since it is the first time ever that such a definition of “victim” been legislated in the world^18^. In this sense, this legislation is based on evidence verifying that on many occasions, helping, protecting, and defending victims of this type of violence generates new violence differentiated from the previous violence. For this reason, it is known by the term “second order,” which links this victim with the one who first suffers the act or the illicit situation. Until now, these people were not recognized as victims under any legal circumstances. Their conscious and voluntary action was left in legal limbo. To date, the norm has focused on who is a direct victim of violence. However, second-order harassment implies a manifestation of violence that produces different and autonomous effects from those suffered by the direct victim. The person who conducts this harassment may be the same person or another person; moreover, the harassment may or may not have a more collective and institutional component, and it can generate a type of reprehensible consequence different from what the direct victim experienced. The individualization of this behavior, its express classification in the norm, deserves to be highlighted since it will encourage people to support and will undoubtedly contribute to the eradication of this type of violence.

## Discussion

The categorization of second-order violence and its broad concept of second-order sexual harassment undoubtedly indicates a way to pass laws equivalent to social realities. Laws usually follow social problems; however, institutions might be able to provide a guiding role. Having shown that support empowers victims and allows them to come forward, the need to dedicate efforts to overcome SOSH is apparent. Research has shown that sexual harassment is a problem that must addressed from different aspects since it can occur in several spheres. Addressing it within various contexts, such as academia, leads to its later application to other areas, such as politics, media, and schools. The scientific literature identifies this concept as a key topic that must be taken into account in public policies to give protection to the people who can overcome silence (Madrid et al., 2020; Melgar et al., 2020).

While scientific literature tends to be somewhat vague on defining a profile of “victim” or a profile of “harasser” in some spheres, they do agree on patterns for victims’ support, protection and empowerment. What we are truly considering and undertaking here is extending the proven system of supporting survivors through informal instruments by highlighting the benefits of formal mechanisms. In this line, an uncomfortable space in terms of working and studying creates a barrier for people—especially for women—in academia, business, and other fields, such as healthcare. Approaching sexual harassment contributes to overcoming inequalities and promoting women’s leadership (Kalaitzi et al., 2017). In the same vein, the contribution of this article opens the path for social policy implications as mechanisms and policies for working with survivors and addressing their needs, which may include the SOSH perspective.

In other words, access to justice would be enabled and fear of reporting may decrease. Responding to victims of SOSH means getting more women and more people to file more complaints and helping them move forward while diminishing the impunity of the aggressors. SOV law provides a legal response to a social problem that has not been properly covered and promoted helplessness among women and granted impunity to aggressors. The current debate claims to change laws and update them at the current moment. Evidence-based legislation is also needed, and there is not a better moment than the current one to address this topic properly. Within this framework, such legislation is evidence that SOSH occurs. Legislating SOSH provides an answer to its need according to the associated evidence. In this line, Catalan legislation will contribute to decreasing gender-based violence by enabling access to justice for direct and second-order victims while overcoming its lack of regulation, which dramatically obstructs the protection and response in GBV cases.

## Conclusions

While much attention is given to direct victims, the reality of second-order sexual harassment victims is rarely studied. Recognizing sexual harassment involves analyzing SOSH and its implications. A satisfactory legal consideration for this concept embraces the importance of including SOV in legislative concepts and thus supporting a more egalitarian society. The current legal framework responds to this deficiency by enacting a law that recognizes the need for relief for second-order victims. Thus far, bystander interventions, networks of support, and SOSH have led to the current legislation of the second-order violence concept. Even if the reality of SOSH emerges in the university context, both in the USA and in Spain, harassment and criticism against those who intervene to support direct victims occur in different contexts, such as the media, leisure time, or workplace. Support is needed in all contexts, for everyone (Kelly et al., 2020). Why this support is not provided is the question answered by this article.

The pioneer Catalan legislation described in this article contributes to eradicating sexual harassment and bringing SOSH closer to the spectrum of gender studies, which extends beyond the current reality of direct victims. It is through this approach that the mechanisms by which a social claim can become law are justified. In 2017, even greater social mobilization began, exemplified to some extent in campaigns such as #Metoo or #TimesUp, which led to a generation of rejection against sexism and sexual violence in all areas and addressing the deep silence existing around it. Currently, society faces a historical opportunity to take advantage of this crucial period of breaking the silence to continue taking steps towards achieving more equal institutions and social spaces as we all have once dreamed.

Addressing SOSH is also devoted to UN Sustainable Development Goal number 5 on Gender Equality, in which goal 5.2 includes eliminating all forms of violence against women (UN-SDG, 2017). This reality also generates social responsibility to build a framework of protection so that every person and every survivor has a support network to count on. This new approach provides legitimacy for social movements against sexual violence while legitimizing the *Struggle for Law*, in Ihering’s words (2003:1878), by establishing it in a way so that social struggle is required to achieve the existence of law. In the same vein, in his *Theory of Legal Norms*, Norberto Bobbio (1958) offers a relevant contribution that relates history to the set of norms that follow and integrate each other, and they share common features of being propositions aimed at influencing the behaviors of groups and individuals. The law not only “goes behind society” by regulation, but the norm itself generates patterns of behavior towards one side or the other.

Many advancements have been made, and further research on harassment victims’ protection is needed. Thus, many more studies are yet to come. This paper becomes the starting point of many other scientific contributions that will lead to much more legislation and advancements against sexual violence. Many harassment situations remain unreported and therefore unknown. Moreover, empowering survivors and encouraging them to report and contributing to turning bystanders into active upstanders overall means advancing the fight to eradicate gender-based violence from a safe and inclusive context. Legislating second-order violence ensures that actions that were previously only considered unethical are now against legal regulations.

## Footnotes

1 Title IX defends the following: *every student has an equal right to education, regardless of their gender identity and expression*.

2 For more information, see the Legal Portal of the Government of Catalonia’s official Webpage. Retrieved from: https://portaljuridic.gencat.cat/ca/pjur_ocults/pjur_resultats_fitxa?action=fitxa&documentId=491383#fragment-1407595

3 Directive 2006/54/EC of the European Parliament and the Council on July 5, 2006 on the application of the principle of equal opportunities and equal treatment for men and women in employment and occupation matters (recast). DOUE L 204/23, 26.7.2006; and Council Directive 2004/113/EC on December 13, 2004, which applies the principle of equal treatment between men and women to access to goods and services and their supply. DOUE L 373/37, 21.12.2004.

4 BOE number 71 on March 23, 2007.

5 BOE number 101 of April 28, 2015.

6 As an “indirect” victim in the cases of death or disappearance of a person who has been caused directly by a crime, except in the case of those responsible for the events, the following considerations are taken into account: 1. Your spouse (not legally separated or in fact) and the children of the victim or of the spouse (not legally or de facto separated) who at the time of the death or disappearance of the victim lived with them; to the person who until the moment of death or disappearance had been united to her by an analogous relationship of affection and to her children who at the moment of the death or disappearance of the victim lived with her; to her parents and relatives in a direct or collateral line within the third degree who are under her care and to the people subject to her guardianship or curatorship or who are under her foster care. 2. In the absence of the above, to the other relatives in a straight line and to their siblings, preferably, among them, the one who will hold the legal representation of the victim.

7 It replaces Framework Decision 2001/220/JHA of the Council and expands in this sense the concept of victim to include not only natural persons as direct victims but also family members.

8 Op.cit., p.37-38. This broad reference to the consideration of victim was described in the draft of the Draft Criminal Procedure Code (art. 59 ACPP)

9 For more information, see: https://www.youtube.com/watch?v=fjjk1aeEOJQ&t=2s

10 For more information, see: https://eldiariofeminista.info/2020/12/23/impacto-social-tras-cuatro-anos-delas-primeras-jornadas-deacoso-sexual-desegundo-orden/

11 For more information, see: https://eldiariofeminista.info/2020/12/18/avance-historico-en-la-lucha-contra-la-violencia-degenero-desde-los-movimientos-sociales/

12 Approved in the 64th plenary session, December 16, 2005. It is preceded by the Declaration on the fundamental principles of justice for victims of crime and abuse of power, resulting from the debates of the Seventh United Nations Congress on Crime Prevention and Treatment of the Offender.

13 The aforementioned Declaration of November 29, 1985, also considered a victim in the same terms: The term “victim” also includes, where appropriate, the immediate family or dependents of the direct victim and persons who have suffered *harm in intervening to assist victims in distress or to prevent victimization (italics ours).* Retrieved from: https://www.ohchr.org/en/professionalinterest/pages/victimsofcrimeandabuseofpower.aspx

14 For more information, see: https://en.wikipedia.org/wiki/Theo_van_Boven

15 Redress Trust, *Implementing victims’ rights: A Handbook on the Basic Principles and Guidelines on the Right to a Remedy and Reparation*, March 2006, p. 19: “Finally, the definition of “victim” also includes persons who have suffered harm in intervening to assist victims in distress or to prevent victimization. It is very common for human rights lawyers or doctors assisting victims of human rights abuses to be targeted. When considering the harm suffered, the context in which the violations took place needs to be taken into account to define the victimization and right to reparation. For example, a lawyer defending victims of gross abuses might be ill-treated or physically abused, or intimidated through phone calls; written threats; random questionings; short detentions; ‘mistaken’ arrests. In these cases, the distress, harm and loss suffered needs to be considered in the context of the overall pattern of serious violations of international human rights or humanitarian law.”

16 Clara Sandoval-Villalba, “The Concepts of “Injured Party” and “Victim” of Gross Human Rights Violations in the Jurisprudence of the Inter-American Court of Human Rights: A Commentary on their Implications for Reparations”. In Reparations for Victims of Genocide, War Crimes and Crimes Against Humanity; systems in place and crimes against humanity; systems in place and systems in the making, 2009, pp. 248-264, performs a historical evolution of the concept of “victim” in jurisprudence.

17 For more information, see the document of the 5/2008 law modification into the 17/2020 Catalan Law for the women right to eradicate sexist violence: https://portaldogc.gencat.cat/utilsEADOP/PDF/8303/1828756.pdf

18 For more information, see: https://eldiariofeminista.info/2020/12/18/innovacion-legislativa-a-nivel-mundial/
